# Surgical treatment for pulmonary metastases from esophageal carcinoma after definitive chemoradiotherapy: Experience from a single institution

**DOI:** 10.1186/1749-8090-6-135

**Published:** 2011-10-12

**Authors:** Yoshiki Kozu, Hiroshi Sato, Yasuhiro Tsubosa, Hirofumi Ogawa, Hirofumi Yasui, Haruhiko Kondo

**Affiliations:** 1 Division of Thoracic Surgery, Shizuoka Cancer Center, Shizuoka, Japan; 2 Division of Esophageal Surgery, Shizuoka Cancer Center, Shizuoka, Japan; 3 Division of Therapeutic Radiology, Shizuoka Cancer Center, Shizuoka, Japan; 4 Division of Gastrointestinal Medicine, Shizuoka Cancer Center, Shizuoka, Japan

**Keywords:** esophageal carcinoma, definitive chemoradiotherapy, complete response, pulmonary metastases, surgical treatment

## Abstract

**Background:**

Surgical treatment for pulmonary metastases is known to be a safe and potentially curative procedure for various primary malignancies. However, there are few reports regarding the prognostic role of surgical treatment for pulmonary metastases from esophageal carcinoma, especially after definitive chemoradiotherapy (CRT).

**Methods:**

We retrospectively reviewed 5 patients who underwent surgical treatment for pulmonary metastases from esophageal carcinoma at our institution. The primary treatment for esophageal carcinoma was definitive CRT, and a complete response (CR) was achieved in all patients.

**Results:**

The surgical procedure for pulmonary metastases was wedge resection, and pathological complete resection was achieved in all 5 patients. The disease free interval after definitive CRT varied from 7 to 36 months, with a median of 19 months. There were no perioperative complications, but postoperative respiratory failure occurred in 1 patient. The postoperative hospital stay varied from 4 to 7 days, with a median of 6 days. Three patients are now alive with a good performance status (PS) and are disease free. The other 2 patients died of primary disease. The overall survival after surgical treatment varied from 20 to 90 months, with a median of 29 months.

**Conclusions:**

Surgical treatment should be considered for patients with pulmonary metastases from esophageal carcinoma who previously received CRT and achieved a CR, because it provides not only a longer survival, but also a good postoperative PS for some patients.

## Background

Surgical treatment for pulmonary metastases is known to be a safe and potentially curative procedure for various epithelial tumors, germ cell tumors, and sarcomas. For example, in the case of surgical treatment for pulmonary metastases from colorectal cancer, the reported overall 5-year survival rate is approximately 40% [1-5]. Even if colorectal metastases extended to both the lungs and liver, surgical treatment can still provide a survival benefit for properly selected patients.

On the other hand, there are few reports regarding the role of surgical treatment for pulmonary metastases from esophageal carcinoma [6,7]. Esophageal carcinoma can cause systemic spread at an early stage [8], and esophageal pulmonary metastases are often detected as multiple lesions, accompanied with other sites of metastasis. Reflecting these lethal propensities of esophageal carcinoma, surgical treatment for pulmonary metastases from esophageal carcinoma is rarely performed. This is presumably the main reason why there have so far been few reports.

Nevertheless, the lungs are one of the most frequent sites of metastases from esophageal carcinoma, and it is of paramount importance to conduct further investigations to identify an effective therapeutic modality for pulmonary metastases from esophageal carcinoma. In this article, we report our institutional experience with surgical treatment for pulmonary metastases from esophageal carcinoma after definitive chemoradiotherapy (CRT).

## Methods

After obtaining institutional review board approval, we retrospectively reviewed a total of 5 patients who underwent surgical treatment for pulmonary metastases from esophageal carcinoma at the Shizuoka Cancer Center, Shizuoka, Japan, between September 2002 and December 2010. All patients had received definitive CRT for esophageal carcinoma as the primary treatment, and a complete response (CR) was achieved. Follow-up radiological examinations were performed using the following method unless the patient presented with clinical symptoms; chest X-rays at every examination in the outpatient department, and computed tomography (CT) scans of the chest and abdomen every 3-6 months. The median follow-up period was 29 months (range, 20-90). During the follow-up period, newly detected round-shaped pulmonary lesions on radiological examination were regarded as metastases from esophageal carcinoma. The selection criteria for surgical treatment of the pulmonary metastases from esophageal carcinoma were as follows; (i) the patient has a performance status (PS) of 0 or 1 based on the ECOG scale and can tolerate surgery, (ii) there is radiological evidence of the resectability of all pulmonary metastases, (iii) the primary esophageal carcinoma is controlled, and (iv) there are no metastatic lesions other than those in the lungs. All patients met these criteria when pulmonary metastases were detected, and therefore underwent surgical treatment. The pre-treatment clinical staging for esophageal carcinoma was based on the 2009 International Union Against Cancer TNM classification. The histological diagnosis of the resected pulmonary specimens was made by at least 2 experienced pathologists. After confirming not only the histological similarity between the resected pulmonary specimens and the esophageal carcinoma, but also the unlikelihood of a second primary lung cancer, they diagnosed the resected pulmonary specimens to be metastatic. We analyzed the clinicopathological data of all patients in detail regarding esophageal carcinoma, pulmonary metastases, surgical procedure, perioperative complications, postoperative hospital stay, disease free interval (DFI), and overall survival (OS). The DFI was calculated as the period from the start of CRT until initial detection of pulmonary metastases on the follow-up CT-scan. The OS was calculated as the period from pulmonary metastasectomy until death or the date of the last follow-up evaluation.

## Results

Our study included 5 males with a median age at surgery of 68 years (range, 55-74). Esophageal carcinoma was located in cervical esophagus (Ce) in 3 patients, and in the upper thoracic esophagus (Ut) in 2 patients. The histological type of esophageal carcinoma was squamous cell carcinoma (SCC) in all patients. The pre-treatment clinical stage of the esophageal carcinoma was IIIA and IIIC in 1 and 4 patients, respectively. The reason for the choice of definitive CRT rather than surgery as the primary treatment for esophageal carcinoma was unresectability due to invasion to the subclavian artery in 1 patient, and refusal of surgery by 4 patients. CRT consisted of 2 cycles of cisplatin 40 mg/m^2 ^on days 1 and 8 and continuous infusion of 5-fluorouracil 400 mg/m^2 ^on days 1 to 5 and 8 to 12, with concurrent irradiation of 60 Gy in 30 fractions. In 1 patient, nedaplatin was administered instead of cisplatin because of the patient's renal function. The DFI varied from 7 to 36 months, with a median of 19 months. Before detection of the pulmonary metastasis, one patient underwent a total pharyngolaryngoesophagectomy for local recurrence. Chemotherapy with docetaxel (DOC) was delivered prior to pulmonary resection in 1 patient, resulting in progressive disease (PD). The surgical procedure used for pulmonary metastases was wedge resection, and pathological complete resection was achieved in all patients. We omitted hilar and mediastinal lymph node dissection during surgery, because there were no enlarged or suspicious lymph nodes noted on the preoperative radiological examination. All resected pulmonary specimens were diagnosed as metastases from esophageal carcinoma. The number of pulmonary metastasis was 1 in 3 patients, and 2 in 2 patients. Except for 1 micrometastasis, the diameter of the pulmonary metastasis varied from 6 to 20 mm, with a median of 12 mm. Respiratory failure occurred postoperatively in 1 patient. The postoperative hospital stay varied from 4 to 7 days, with a median of 6 days. During the follow-up period, another pulmonary metastasis developed in 1 patient, and pulmonary resection was performed again. The OS varied from 20 to 90 months, with a median of 29 months. Three patients are currently alive without recurrence, and the other 2 patients died of primary disease. The details of the patients' backgrounds are shown in Tables 1 and 2.

**Table 1:** Clinicopathological features of the 5 patients with esophageal carcinoma

Patient					
	**1**	**2**	**3**	**4**	**5**

Age	69	59	68	74	55
Gender	M	M	M	M	M
Location	Ce	Ce	Ce	Ut	Ut
Clinical stage (TNM)	IIIC (T4bN0M0)	IIIC (T4bN1M0)	IIIC (T4bN1M0)	IIIA (T3N1M0)	IIIC (T4bN1M0)
Histology	SCC	SCC	SCC	SCC	SCC
CRT regimen	FP + RT	FP + RT	FP + RT	NF + RT	FP + RT
Therapeutic effect of CRT	CR	CR	CR	CR	CR
First recurrence site	Local^a^	Lung	Lung	Lung	Lung

M, male; Ce, cervical esophagus; Ut, upper thoracic esophagus; SCC, squamous cell carcinoma; CRT, chemoradiotherapy; FP, 5-fluorouracil plus cisplatin; NF, nedaplatin plus 5-fluorouracil; RT, radiotherapy; CR, complete response

^a ^a total pharyngolaryngoesophagectomy was performed

**Table 2:** Clinicopathological features of the 5 patients regarding pulmonary metastases and survival

Patient					
	**1**	**2**	**3**	**4**	**5**

DFI (months)	36	20	7	8	19

Number of metastases	1	1	2 (1)^b^	1	2

Diameter (mm)	9	5	20, 15 (15)^c^	20	6^i^

Treatment prior to surgery	None	None	DOC	None	None

Surgical procedure	Wedge resection	Wedge resection	Wedge resection^d^	Wedge resection	Wedge resection

Lymph node dissection	Not done	Not done	Not done^e^	Not done	Not done

Curability	Complete resection	Complete resection	Complete resection^f^	Complete resection	Complete resection

Perioperative complications	None	Respiratory failure	None^g^	None	None

Postoperative hospital stay (days)	6	7	7 (6)^h^	4	5

OS (months)	41	29	28	90	20

Survival	Alive	Dead	Alive	Dead	Alive

DFI, disease free interval; DOC, docetaxel; OS, overall survival

^b,c,h ^The number in parentheses indicates the outcome of the second pulmonary resection

^d,e,f,g ^The common outcome from both the first and second pulmonary resections

^i ^Another pulmonary micrometastasis was detected by pathological examination

## Patient descriptions

### Patient 1

A 69-year-old male was diagnosed with esophageal SCC in the Ce. A pre-treatment CT-scan revealed direct invasion to the trachea (clinical stage T4bN0M0). He chose CRT as the primary treatment, and a CR was achieved. Six months after the start of CRT, a local recurrence developed, so we performed salvage surgery via total pharyngolaryngoesophagectomy with reconstruction by the free jejunum. On a follow-up CT-scan, a solitary pulmonary metastasis was detected 30 months after the salvage surgery. Pulmonary wedge resection was performed, and pathological complete resection was achieved. The patient's postoperative hospital stay was 6 days. He has been disease free for 41 months after pulmonary resection, and was doing well in a check-up performed in the outpatient department of our institution.

### Patient 2

**Figure 1:**
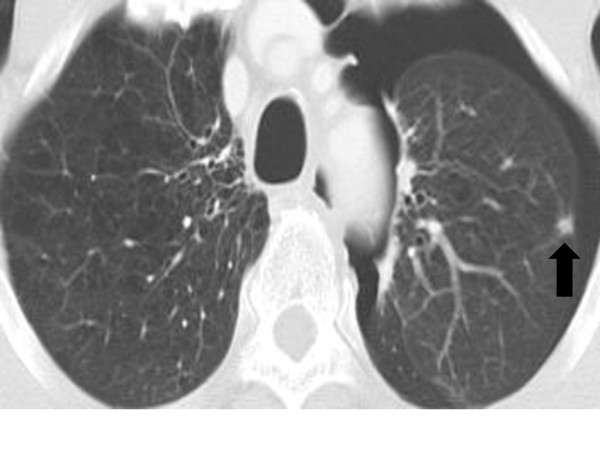
**A follow-up CT scan showing a left pneumothorax, which developed secondary to pulmonary metastasis (arrow)**.

A 59-year-old male was diagnosed with esophageal SCC in the Ce. A pre-treatment CT-scan revealed direct invasion to the trachea (clinical stage T4bN1M0), and bilateral recurrent nerve paralysis was also detected by a laryngeal fiberscope. He chose CRT as the primary treatment, and a CR was achieved. Twenty months after the start of CRT, a follow-up CT-scan revealed a left pneumothorax which had developed secondary to pulmonary metastasis (Figure 1). The air leak persisted even after treatment with chest tube drainage. Subsequently, pulmonary wedge resection was performed, and pathological complete resection was achieved. Postoperatively, respiratory failure caused by bilateral recurrent nerve paralysis occurred, requiring re-intubation and tracheostomy. He recovered well soon after these procedures. The patient's postoperative hospital stay was 7 days. Four months later, a local recurrence developed, and he received a total of 6 cycles of cisplatin and 5-fluorouracil. The therapeutic effect resulted in PD, with the appearance of new lung metastasis. He died of disease 29 months after pulmonary resection.

### Patient 3

A 68-year-old male was diagnosed with esophageal SCC in the Ce. A pre-treatment CT-scan revealed direct invasion to the trachea (clinical stage T4bN1M0). He chose CRT as the primary treatment, and a CR was achieved. Seven months after the start of CRT, a follow-up CT scan revealed 2 pulmonary metastases located in the right upper and lower lobes, and a total of 10 courses of DOC was delivered. However, the pulmonary metastases enlarged, resulting in PD. Subsequently, pulmonary wedge resection was performed, and pathological complete resection was achieved. Twenty-five months later, a contralateral pulmonary metastasis developed, and pulmonary wedge resection was performed again. The postoperative hospital stay was 7 and 6 days after the first and second pulmonary resections, respectively. He has been disease free for 3 months after the second pulmonary resection, and was doing well in a check-up performed in the outpatient department of our institution.

### Patient 4

A 68-year-old male was diagnosed with esophageal SCC in the Ut. The clinical stage was T3N1M0 based on the pre-treatment radiological examination. He chose CRT as the primary treatment. In this case, nedaplatin was administered instead of cisplatin, because the patient had undergone a left nephrectomy due to ureteral carcinoma. Although a CR was achieved, a follow-up CT-scan revealed a solitary pulmonary metastasis 8 months after the start of CRT. Pulmonary wedge resection was performed, and pathological complete resection was achieved. The patient's postoperative hospital stay was 4 days. Nineteen months later, radical resection of a bone (rib) metastasis was performed. Multiple metastases in the local site, pleura and liver gradually developed, and he died of disease 90 months after pulmonary resection.

### Patient 5

**Figure 2:**
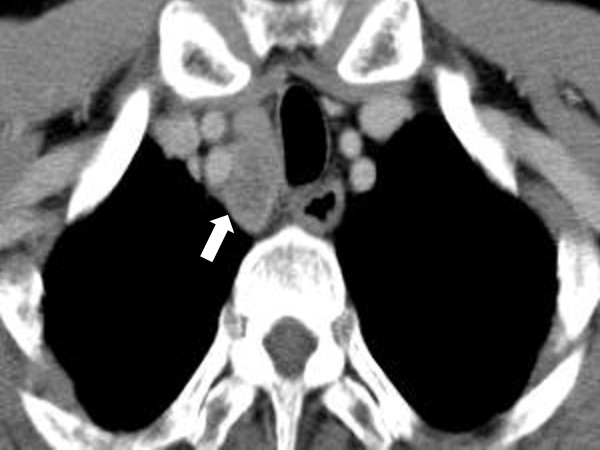
**A pre-treatment CT-scan showing metastatic lymph node invasion to the right subclavian artery (arrow)**.

A 55-year-old male was diagnosed with esophageal SCC in the Ut. A pre-treatment CT scan revealed that a metastatic lymph node had invaded to the right subclavian artery (clinical stage T4bN1M0, Figure 2). CRT was therefore administered as the primary treatment, and a CR was achieved. Nineteen months after the start of CRT, a follow-up CT-scan revealed a solitary pulmonary metastasis. Pulmonary wedge resection was performed, and the pathological examination revealed another pulmonary micrometastasis within the resected specimen which was not detected by the preoperative radiological examination. Pathological complete resection of these 2 metastases was achieved. The patient's postoperative hospital stay was 5 days. He has been disease free for 20 months after pulmonary resection, and was doing well in a check-up performed in the outpatient department of our institution.

## Discussion

In this article, we reviewed our institutional experience with 5 patients who underwent surgical treatment for pulmonary metastases from esophageal carcinoma. A major characteristic of this article is that the primary treatment for esophageal carcinoma was confined to definitive CRT, and a CR was achieved in all patients.

The reported 5-year survival rate of those who are treated with definitive CRT for esophageal cancer is 22.9% in Japan [9], and this procedure is considered to be promising as a primary treatment, although substantial toxicities are associated with the treatment [10]. While surgery still remains a standard curative treatment for resectable esophageal cancer, definitive CRT has become a prevalent alternative as a nonsurgical treatment for unresectable esophageal carcinoma or potentially resectable esophageal carcinoma when patients refuse surgery. Some retrospective studies have reported that CRT showed comparable therapeutic effects as esophagectomy [11,12].

In the case of local recurrence of esophageal carcinoma after definitive CRT, salvage esophagectomy is reported to provide a long survival for some patients, like our current patient 1, at the cost of high rates of morbidity and mortality [13,14]. In contrast, little is known about the impact of surgical treatment for pulmonary metastases from esophageal carcinoma after definitive CRT. This is mainly because the metastases are often detected as multiple lesions and accompanied by metastases to other sites. Only a fraction of cases are therefore considered to be suitable for surgical treatment. As the lung is one of the most common distant recurrence sites of esophageal carcinoma, it is necessary to accumulate cases of the surgical treatment for pulmonary metastases from esophageal carcinoma to elucidate its efficacy.

A previous report indicated that solitary pulmonary metastasis from esophageal carcinoma was a favorable indicator for surgical treatment [6]. In this article, 3 patients with solitary pulmonary metastasis also showed a long survival. It is also worth noting that the other 2 patients with 2 pulmonary metastases are still alive and disease free. Surgical treatment can therefore be beneficial even for patients with more than one pulmonary metastasis from esophageal carcinoma.

The DFI is generally recognized as a significant prognostic factor after surgical treatment for pulmonary metastases from various primary cancers [15,16]. Shiono et al. reviewed 49 surgical cases of pulmonary metastases from esophageal carcinoma. The primary treatments were surgery alone (53%), radiotherapy alone (4%), combined modality therapy (32%), and unknown (10%). They suggested that a DFI greater than 12 months was a favorable clinical factor significantly related to OS [7]. In this article, the DFI in patient 4 was relatively short, at 8 months, compared to the median DFI (19 months), however, that patient's OS was 90 months, which was the longest of all of the patients. Therefore, such patients should be kept in mind, and the possibility of surgical treatment even in those who develop an early recurrence should not be excluded.

The advantages of surgical resection over chemotherapy for pulmonary metastases are a shorter hospital stay, fewer treatment-related complications, a better PS after treatment, and certainty of tumor removal. For metastatic esophageal carcinoma, the standard chemotherapeutic regimen with cisplatin and 5-fluorouracil yields modest response rates of 25 - 33%, but a CR is rarely achieved [17]. The benefit of chemotherapy has yet to be proven. Moreover, chemotherapy-related complications such as neurological, haematological, and renal toxicities are significant, leading to a worse PS compared to untreated patients [18]. On the other hand, surgical treatment for pulmonary metastases is a safe and well established procedure for properly selected patients. All of our present patients were able to undergo pathological complete resection by pulmonary wedge resection, and were discharged from the hospital within 7 days after surgery with a good PS. Even after definitive CRT, surgical treatment for pulmonary metastases from esophageal carcinoma seems to be justified.

We were able to demonstrate that the procedure has prognostic implications, because it led to a median OS of 29 months (range 20-90), whereas the previously reported median OS were 24 and 27 months [6,7]. In the previous reports, definitive CRT was not administered as the primary treatment for esophageal carcinoma. Although only 5 cases were included in this study, we believe that surgical treatment for pulmonary metastases from esophageal carcinoma can provide a long survival for those whose primary treatment was definitive CRT and who achieved a CR from that treatment. Taken together, our findings indicate that surgical treatment can presumably be used an alternative to systemic chemotherapy in treating pulmonary metastases from esophageal carcinoma, if the patients meet the above described criteria.

## Conclusions

Surgical treatment should be taken into consideration for patients with pulmonary metastases from esophageal carcinoma who previously received CRT and achieved a therapeutic CR, because it can provide not only a longer survival, but also a good postoperative PS for some patients.

## Consent

Written informed consent was obtained from the patients for publication of this case report and accompanying images. A copy of the written consent is available for review by the Editor-in-Chief of this journal.

## Abbreviations

CRT: chemoradiotherapy; CR: complete response; CT: computed tomography; PS: performance status; DFI: disease free interval; OS: overall survival; Ce: cervical esophagus; Ut: upper thoracic esophagus; SCC: squamous cell carcinoma; DOC: docetaxel; PD: progressive disease.

## Competing interests

The authors declare that they have no competing interests.

## Authors' contributions

HS and YT both conceived of the study, and participated in its design and coordination and helped to draft the manuscript. HO and HY both advised and interpreted of data. HK participated in critical revision of the manuscript. All authors read and approved the final manuscript.
